# Increased absorption and use of nutrients induced by Si is an indicator for tolerance to water deficit in a common bean cultivar cultivated in the field with and without application of K

**DOI:** 10.3389/fpls.2024.1421615

**Published:** 2024-08-09

**Authors:** Gelza Carliane Marques Teixeira, Carlos Vital Gonzalez-Porras, Patrícia Messias Ferreira, Renato De Mello Prado, Kamilla Silva Oliveira, Lívia Tálita da Silva Carvalho, Luiz Fabiano Palaretti

**Affiliations:** Department of Soil Science, São Paulo State University, São Paulo, São Paulo, Brazil

**Keywords:** plant nutrition, water deficiency, water effciency, beneficial element, nutritional efficiency

## Abstract

**Introduction:**

Reduced water content in the soil triggers physiological, biochemical, and morphological damage to plants, aggravated by nutritional deficiency. One possible strategy to mitigate this damage comprises the use of silicon (Si). This study investigated whether Si can mitigate the damage caused by water deficit through nutritional mechanisms in bean plants grown under field conditions. Furthermore, it investigated whether the effectiveness of Si is influenced by water availability in the soil and the Si dose supplied.

**Methods:**

Therefore, two split-plot experiments were carried out: with and without K supply. In both experiments,the treatments comprised a 3 × 4 factorial scheme. Treatments included three water regimes: 80% (no water deficit), 60% (moderate water deficit), and 40% (severe water deficit) of the soil’s water retention capacity. Moreover, they comprised four doses of Si supplied via fertigation—0 kg/ha, 4 kg/ha, 8 kg/ha, and 12 kg/ha—arranged in a randomized block design with four replications.

**Results and discussion:**

The appropriate dose of Si to be applied increased with the severity of the water deficit, with the recommended dose being 6 kg/ha, 7 kg/ha, and 8 kg/ha of Si for adequate water conditions, moderate water deficit, and severe water deficit, respectively.

## 1 Introduction

Climate change has affected crops. Crops are subject to limited water resources and have a longer period of exposure to water deficit (Besharat et al., 2020). Increased exposure time to drought induces secondary stress due to nutritional deficiency, given the need for water to absorb nutrients (Rizwan et al., 2015) and to transport water and nutrients through the xylem to the plant’s aerial part (Ibrahim et al., 2020).

Water deficit can induce nutritional deficiency, aggravating biological damage in the plant. Nutrients, such as potassium (K), have a function associated with leaf water status, increasing leaf area, water use efficiency in stressed plants, and decreasing leaf transpiration (Martineau et al., 2017). Furthermore, K improves net carbon (C) assimilation and the phloem transport of sugars from leaves to roots (Ibrahim et al., 2020). Moreover, it prevents oxidative damage by maintaining reactive oxygen species (ROS) homeostasis and activating antioxidant metabolism enzymes (Ul-Allah et al., 2020), favoring the plant dry matter accumulation rate.

Silicon (Si) is another relevant element that can increase water use efficiency. It is used to alleviate water deficit damage (Vasanthi et al., 2014). Silicon is the second most abundant element after oxygen, with silicon dioxide (SiO_2_) comprising 50%–70% of soil mass (Epstein, 1994). However, in the soil solution available, Si is in the form of monosilicic acid (H_4_SiO_4_) in a low concentration (~1 mmol/L) (Katz et al., 2021). Silicon is absorbed in the form of monosilicic acid by different transport mechanisms (Barreto and Barão, 2023) in the same form in the xylem sap (Mitani et al., 2005). Silicon arrives at the leaves from the loss of water and is concentrated, inducing polymerization and forming amorphous or biogenic silica, constituting 90% of the Si absorbed being present in the cell wall in different structures such as Si-cellulose (Yoshida, 1965). Thus, intercellular structures comprise only a small part of silica at a monomeric level (Woesz et al., 2006).

The beneficial effects of Si in mitigating water deficiency have been reported with special emphasis on physiological mechanisms, such as the decrease in water loss (Teixeira et al., 2022a), due to the formation of phytoliths in cell walls and around the vascular bundle (Mecfel et al., 2007). The limitation of its absorption aggravates the loss of water since dehydrated plants have losses in the imbalance of the osmotic adjustment caused by the accumulation of proline and sugars, an effect partially reversed by Si (Avila et al., 2021). Thus, plants enriched with Si and under water deficit have a positive adjustment in the hydraulic conductivity of the roots (Abbas et al., 2015) and an increase in root growth (Besharat et al., 2020), benefiting water absorption (Teixeira et al., 2020b).

Increased water uptake stimulates nutritional mechanisms by increasing nutrient absorption (Ibrahim et al., 2020) and improving ionic balance, especially in plants under water deficit, which have limited ion contact with the roots (Teixeira et al., 2022a). Studies have indicated that root Si deposition improves cell wall viscoelastic properties, increasing root strength, root length, thickness, volume, biomass, number of lateral roots, nodulation, and nitrogen (N) fixation (Lux et al., 2020; Lukacova et al., 2023). Thus, it increases the volume of roots occupying the soil in depth, favoring nutrient and water absorption.

However, a recent review on Si in water deficit mitigation indicated the need for further studies to better understand this element’s effects on nutrient absorption (Irfan et al., 2023). A pioneering study even indicated that Si increases the rose plant’s tolerance to water deficit by increasing the absorption of some nutrients—phosphorus (P), potassium (K), iron (Fe), zinc (Zn), and copper (Cu) (Hajizadeh et al., 2023). A nutritional factor may largely explain the physiological improvement induced by Si in plants with water restriction. Nevertheless, researchers overlooked it.

The benefit of Si in water-deficit plants may go beyond nutrient uptake, improving the use efficiency of these elements in plant metabolism. Thus, innovative studies reinforce that Si can mitigate water deficit by increasing nutrient use efficiency restricted to C, N, and P. This water deficit mitigation has only been reported in Si-accumulating species, such as first-cut (Teixeira et al., 2020c, 2022b) and second-cut sugarcane (de Oliveira Filho et al., 2021; Costa et al., 2023), and in forage plants (Rocha et al., 2021). The suspicion is that the increase in nutritional efficiency is because Si binds to cell wall components, replacing structural compounds with a high energy cost, such as lignin (Schaller et al., 2012).

This innovative line of research on the nutritional role of Si in plants under water deficit predominates in studies on Poaceae plants (Teixeira et al., 2020a, 2020b, 2021). Furthermore, few studies on Fabaceae show different Si absorption systems (Gonzalez-Porras et al., 2024). Moreover, few studies evaluate simultaneous stress under field conditions. However, it is common for more than one stress to occur (Frew et al., 2018), such as a crop with some drought-related nutritional deficiency.

In this context, the inclusion of Si in agricultural management could collaborate with the rational use of water for irrigation (Fan et al., 2021), both for the direct effects on the improvement of physiological processes in plants under stress and for the synergistic effects of Si with nutrients, such as K. They contribute to the water adjustment in the plant. Silicon also improves the plant’s absorption of K due to the activation of H-ATPase (Mali and Aery, 2008b). Thus, these synergistic effects should benefit plants adequately nourished with K and Si.

Thus, the intensification of crops and Si use in agriculture is necessary to increase the efficiency of applications of this beneficial element. One strategy comprises applying Si via fertigation, which helps reduce silicon polymerization in the soil. By applying doses of less than 20 kg/ha, the plant can absorb the silicon more efficiently, as already observed in sugar cane plantations (de Oliveira Filho et al., 2021; Teixeira et al., 2021), maize (Teixeira et al., 2022a), and forage crops (Rocha et al., 2021). However, the research carried out was limited to controlled environments and pots, and there needs to be more information on the best agronomic dose of Si to be applied through fertigation under field conditions in Brazil.

In this context, balanced plant nutrition can increase nutritional efficiency, especially Si and K. However, this synergy is still unknown, especially in fertigated bean crops. Therefore, it is important to answer the following hypotheses: Si applied in an appropriate dose mitigates damage caused by moderate and severe water deficit based on nutritional mechanisms; if so, K fertilization may interfere with the optimal recommended dose of Si.

In order to test this hypothesis, this study aimed to evaluate the effect of Si doses via fertigation in the absence and presence of K fertilization in three water regimes to support a strategy for efficient water use in an irrigated bean cultivation system. Furthermore, it aimed to unravel the nutritional mechanisms involved.

## 2 Materials and methods

### 2.1 Growing conditions and plant material

**Figure 1:**
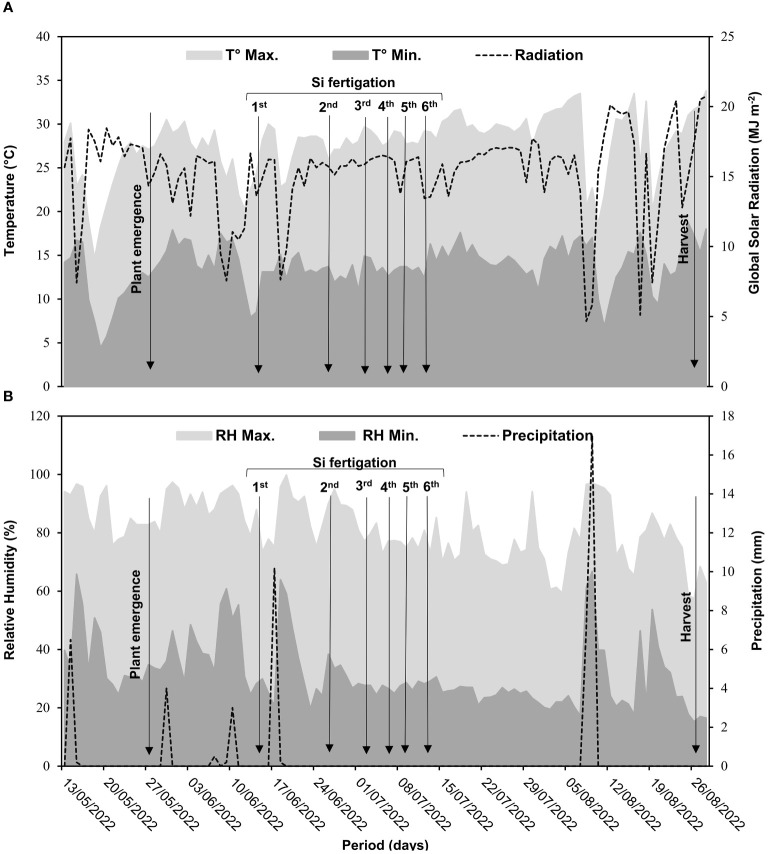
Meteorological data of the experimental area during bean cultivation. Maximum temperature (T° Max.), minimum temperature (T° Min.), and global radiation **(A)**; maximum relative humidity (RH Max.), minimum relative humidity (RH Min.), and precipitation **(B)**.

The experiment was conducted under field conditions at Universidade Estadual Paulista, in Jaboticabal (latitude −21°14′50.7″ and longitude 48°17′01.8″, altitude 546 m), São Paulo, Brazil. Bean cultivation was conducted from May to August 2022. Air temperature, air humidity, global radiation, and rainfall data from the experimental area were recorded daily (**Figure 1**).

Bean seeds of the cultivar Carioca BSR FC 402 (*Phaseolus vulgaris* L.) were used because of their properties: high yield potential, high nutritional value, color uniformity, grain size, resistance to common mosaic, and moderate resistance to anthracnose, rust fungus, and *Fusarium* wilt (Melo et al., 2017). Sowing was conducted with 0.45-m spacing between rows, with a population of 333,000 plants per hectare.

The soil of the experimental area was a eutroferric Red Latosol (Andrioli and Centurion, 1999), which corresponds to Oxisol. Prior to installation, soil samples were collected at depths of 0–20 cm and 20–40 cm for chemical analysis for soil fertility purposes (Raij et al., 2001) to determine the available Si content (Korndörfer et al., 2004) and for granulometric analysis (Camargo et al., 1986). **Table 1** shows the results of the soil’s chemical analysis in the 0–20- and 20–40-cm-deep layers.

**Table 1:** Chemical attributes of 0–20- and 20–40-cm-deep beds.

Prof	pH	OM	P	S	Ca	Mg	K	Al	H + Al	SB^a^	T^b^	V^c^	m^d^	B	Cu	Fe	Mn	Zn
cm	CaCl_2_	g/dm^3^	mg/dm^3^		mmol_c_/dm^3^							%		mg/dm^3^				
0–20	6.2	20.5	59	11	37	17	6.2	1	22.5	60.6	82.9	73	1	0.41	6.4	11	23.6	3.9
20–40	5.6	19	36	13	40	18	6.4	0	23	64	87.2	73	0	–	–	–	–	–

a Sum of bases (SB = Ca^2+^ + Mg^2+^ + K^+^).

b Cation exchange capacity (T = SB + H+Al).

c Basis saturation (V = SB × 100/T).

d Aluminum saturation.

### 2.2 Treatments and experimental design

The treatments comprised a 3 ×4 factorial scheme with three water regimes: 80% [no water deficit (WWD)], 60% [moderate water deficit (MWD)], and 40% [severe water deficit (SWD)] of the soil water retention capacity (WRC). They included four doses of Si supplied via fertigation: 0 kg/ha, 4 kg/ha, 8 kg/ha, and 12 kg/ha. The treatments were evaluated without and with potassium fertilization (50 kg/ha of K_2_O).

The experiment was installed in a subdivided plot scheme (split-plot), with the water regimes considered the primary factor and the Si doses the secondary factor. The treatments comprised a randomized block design with four replications. The experimental plots had dimensions of 2.25 × 6 m, a total area of 13.5 m^2^, and a useful area of 5.4 m^2^.

Silicon doses were determined considering the standard indication of the International Rice Research Institute (IRRI), which recommends using doses of 40 to 60 kg/ha of potassium silicate (15% Si) (equivalent to 6 to 9 kg/ha of Si) (IRRI, 1993). The doses were applied in six installments, at 22 days, 32 days, 38 days, 41 days, 44 days, and 47 days after full emergence (DAE). In other words, each application comprised Si doses of 0 kg/ha, 0.67 kg/ha, 1.33 kg/ha, and 2.0 kg/ha. An irrigation depth of 2.5 mm (2.5 L m^2^) was used to apply Si doses. Thus, Si concentrations in the solution in each application were 0 mmol/L, 0.96 mmol/L, 1.90 mmol/L, and 2.85 mmol/L. Silicon was applied in installments to induce an increase in Si absorption so that the concentrations used in each application were lower than 3.5 mmol/L and, therefore, without risk of polymerization of the element.

The Si source was sodium silicate stabilized with sorbitol (Si = 115.2 g/L, Na_2_O = 60.5 g/L, and 100 mL/L of sorbitol). It was prepared by mixing sodium silicate (Na_2_SiO_3_, Diatom^®^, Sao Paulo, Brazil) and sorbitol (D-Sorbitol C_6_H_14_O_6_, Merck^®^, Darmstadt, Germany) in appropriate proportions. The sorbitol in this source had stabilizing properties that allowed it to provide a higher concentration of monomeric forms of Si, reducing the risks of polymerization of the applied solution (Babiker and Duncan, 1974).

The fertilizations were conducted based on the soil contents and following the recommendation of Ambrosano et al. (1997). Phosphate fertilization was performed by applying a dose of 40 kg/ha of P_2_O_5_ in the form of simple superphosphate in the cultivation line. Nitrogen fertilization was conducted via fertigation using urea, applying 20 kg/ha, 30 kg/ha, 45 kg/ha, and 45 kg/ha of N at 7 DAE, 20 DAE, 31 DAE, and 43 DAE, respectively. For plants cultivated with K fertilization (K1), a dose of 50 kg/ha of K_2_O was applied in the form of KCl by broadcast and manually at 14 DAE. Under the condition of cultivation without potassium fertilization (K0), the plants did not receive a K supply. However, they received the fertilization of the other nutrients.

Soil preparation was conducted by clearing weeds, followed by subsoiling and heavy and light harrowing. Phytosanitary management included control of the screwworm (*Agrotis ipsilon*) at 6 DAE using chlorantraniliprole and Lambda-cyhalothrin (250 mL) + mineral oil (150 mL adjuvant) in 150 L of syrup, applied at a rate of 335 L/ha. A preventive application to control whitefly (*Bemisia tabaci*) and fungi was performed at 14 DAE using thiamethoxam (100 g/L) + fluxapyroxad and pyraclostrobin (150 mL) + mineral oil (150 mL) in 120 L of syrup, applied at a rate of 250 L/ha. Applications to control whitefly were also performed at 30 DAE and 42 DAE using Thiamethoxam (100 g/L) + Mineral oil (150 mL) in 120 L of syrup, applied at a rate of 250 L/ha. Weed control was performed manually until the plants closed the rows.

A self-compensating drip irrigation system was installed in the experimental area, with emitters spaced at 0.5-m intervals and a flow rate of 1.6 L/h (DripNet PC 16250). Each crop line comprised a drip line. Water retention capacity levels in the soil (WRC) were determined by collecting undisturbed soil samples. The water retention curve was constructed through tests on a tension table and Richard’s pressure chamber (Klute, 1986). From the water retention curve, the field capacity (FC) was determined to be 0.41 cm^3^/cm^3^, and the permanent wilting point (PWP) was determined to be 0.13 cm^3^/cm^3^. Using these values, the available water (AW) was calculated as 0.28 cm^3^/cm^3^. Consequently, the total available water capacity (TAW) was determined using AW and the effective root system of the common bean plants, which was 0.4 m. The TAW was calculated to be 112 mm. Based on the TAW value, the soil WRC levels were adjusted to 89.6 mm, 67.2 mm, and 44.8 mm for the water regimes of 80%, 60%, and 40% of WRC, respectively (Webber et al., 2006).

Initially, the plants were maintained under adequate water conditions (80% of WRC), while moderate and severe water deficit levels were imposed during the pre-flowering period (R5). The water volume applied to maintain the soil WRC levels was determined through daily water balance calculations, considering the excess or deficit of water in the soil concerning the WRC of each water regime. The inputs considered in this system included irrigation water and rainfall, while the only water output considered was crop evapotranspiration (ETc), assuming that deep percolation and runoff were negligible.

During the experiment, the volumetric soil moisture (θ) was monitored daily using the time-domain reflectometer (TDR) method (HydroSense II), which is an indirect method. For TDR use, soil samples were initially collected at the same points to determine the soil moisture directly based on mass. The TDR’s precision with soil moisture was considered sufficient with an *R* ^2^ = 0.84 (y = 1.0294x − 3.2562), where the TDR readings underestimated the soil moisture (θ) by 2.2% with a 1% standard deviation. The established soil moisture levels for each water regime were 0.354 cm^3^/cm^3^, 0.283 cm^3^/cm^3^, and 0.212 cm^3^/cm^3^ for the water regimes of 80%, 60%, and 40% of WRC, respectively (Webber et al., 2006).

The irrigations were applied when the water deficit reached the maximum allowable depletion of available water in the soil. For the irrigation scheme recommended by the Food and Agriculture Organization of the United Nations (FAO) (Allen et al., 2006), without water deficit, the experimental plots were irrigated when 45% of the available water in the soil was depleted, resulting in a soil moisture (θ) of 0.2864 cm^3^/cm^3^ and water storage of 49.58 mm. The depletion factors for the moderate and severe deficit treatments were 60% and 80%, with soil moisture (θ) at 0.242 cm^3^/cm^3^ and 0.184 cm^3^/cm^3^ and water storage of 26.88 mm and 13.44 mm, respectively.

The plants were cultivated throughout the entire phenological and morphological cycles. Physiological and nutritional analyses were conducted during the pod formation stage (R7). The analyses were determined by collecting samples from 10 plants per plot within the useful area (5.4 m^2^ of each experimental plot).

### 2.3 Analyses

#### 2.3.1 Dry matter production

The plants’ shoots were collected and washed in running water, detergent solution (0.1% v/v), and HCl solution (0.3% v/v) and later in deionized water. The plant material was dried in a forced air circulation oven (TE-394/3-MP, Tecnal, Ourinhos, Brazil) (65°C ± 5°C) until reaching constant matter. Each experimental unit comprised 10 plants randomly collected from the useful area of each plot.

#### 2.3.2 Accumulation of Si, C, N, P, K, Ca, Mg, S, and micronutrients in the shoot

After drying, the plant tissue samples collected to determine the dry mass were ground in a Willey-type mill and used to determine the Si and nutrient contents. The Si concentration was obtained by extracting the element with hydrogen peroxide and sodium hydroxide solution in a forced air circulation oven (TE-394/3-MP, Tecnal, Brazil) at 120°C ± 5°C (Kraska and Breitenbeck, 2010). Reading was performed by colorimetry with ammonium molybdate, oxalic acid, and HCl using a spectrophotometer (B442, Micronal, Sao Paulo, Brazil) at 410 nm (Korndörfer et al., 2004).

The foliar concentrations of P, K, Ca, Mg, S, Mn, Fe, Zn, and Cu were determined from the digestion of the samples with perchloric acid and nitric acid (2:1) (Bataglia et al., 1983). The reading of K, Ca, Mg, Fe, Zn, and Cu was performed by atomic absorption spectrophotometry following the manufacturer’s instructions. The P concentration was determined by colorimetric reaction with ammonium molybdate and ammonium vanadate with reading in a spectrophotometer at 420 nm. The S concentration was determined by a reaction induced with SEED acid and barium chloride with a reading in a spectrophotometer at 420 nm. The P and S concentrations were determined following the methodology proposed for both elements (Bataglia et al., 1983).

The N concentration was determined by digesting the samples with concentrated sulfuric acid. Then, the samples were subjected to distillation and titration with sulfuric acid (Bataglia et al., 1983). The C concentration was determined by dry combustion at 1,000°C using a LECO Truspec CHNS elemental analyzer calibrated with a LECO wheat standard (502–278, Sector Joseph, MI, USA) (C = 45%).

The accumulation of elements in the shoot was determined from the ratio of the concentrations obtained with the dry matter of the plants using the equation below:


$$\begin{array}{c} Silico\text{n }an\text{d }nutrien\text{t }accumulatio\text{n }\left(\text{g }o\text{r }m\text{g }pe\text{r }plant\right)\\ =\frac{Concentratio\text{n }\left(\text{g }o\text{r }m\text{g }kg^{-1}\right)}{Dr\text{y }matte\text{r }\left(k\text{g }pe\text{r }plant\right)} \end{array}$$

#### 2.3.3 Use efficiencies of C, N, P, K, Ca, Mg, and S

The use efficiencies were determined using the equation below (Fageria and Baligar, 2005):


$$Us\text{e }efficienc\text{y }\left(g^{2}g^{-1}\right)=\frac{Shoo\text{t }dr\text{y }matter^{2}\;\left(g\right)}{Accumulatio\text{n }o\text{f }nutrient\text{s }\left(\text{g }pe\text{r }plant\right)}$$

### 2.4 Statistical analysis

The data underwent a bidirectional analysis of variance by the F-test (*p* ≤ 0.05) after the normality and homoscedasticity of variances (Bartlett’s test and the Shapiro–Wilks W test). Quantitative data corresponding to Si doses were analyzed using mathematical regression models. Levels of significance of the mathematical regression models are represented by Pearson’s (*R* ^2^) linear correlation test. Qualitative data corresponding to water regimes were analyzed using Tukey’s honestly significant difference average comparison test (*p* ≤ 0.05).

Principal component analysis (PCA) was performed using the correlation matrix, including a hierarchical cluster analysis using Euclidean distance as the similarity coefficient. Furthermore, a correlation network was used to graphically express the functional relationship of Pearson’s correlation coefficient estimates between variables. Thus, the proximity between the traits was proportional to the absolute value of the correlation between them. Positive correlations were highlighted in green, while negative ones were represented in red. Statistical analyses were performed using the R programming language (version 4.3.1, R Core Team).

## 3 Results

**Figure 2:**
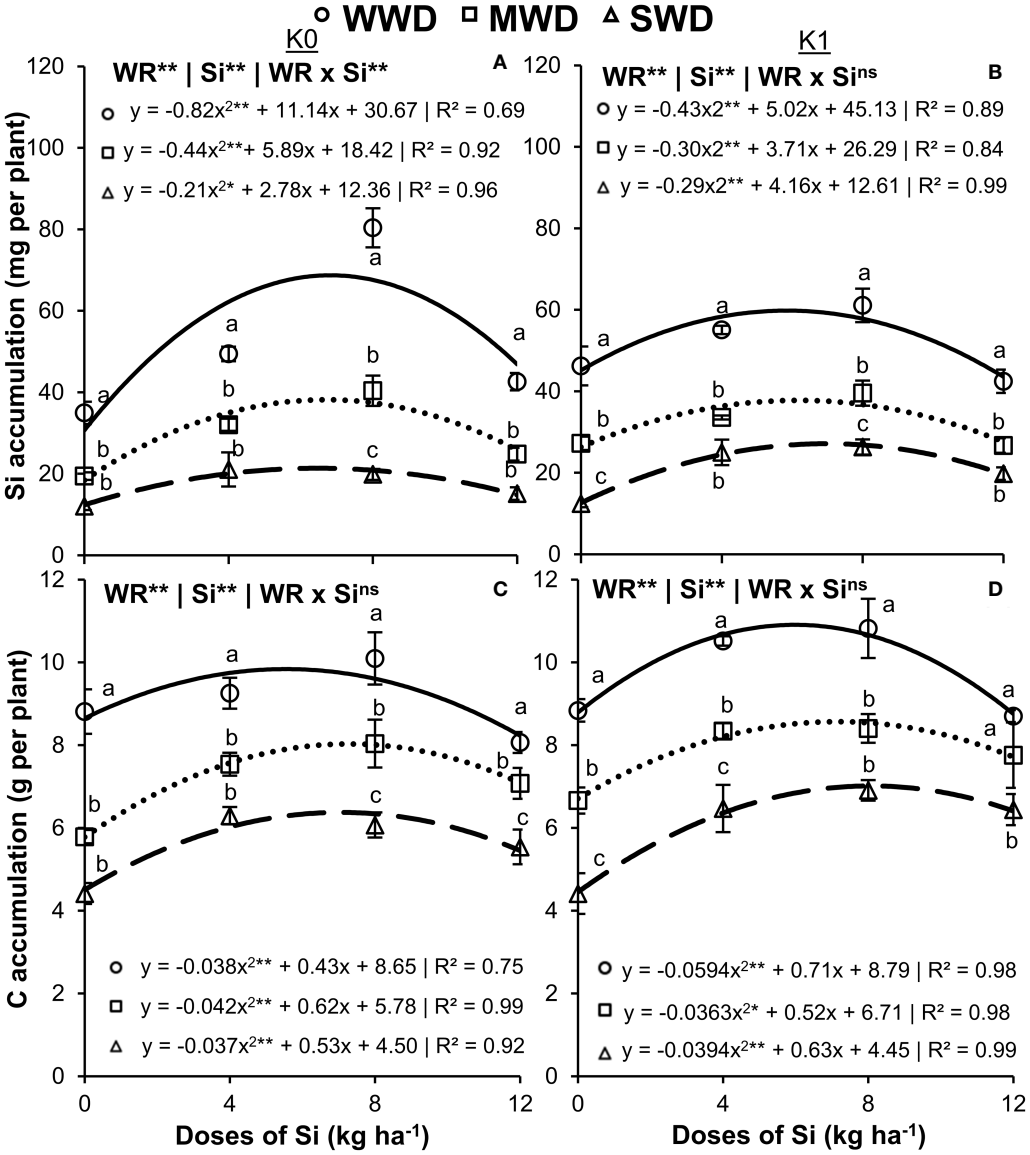
Accumulation of silicon (Si) **(A, B)** and carbon (C) **(C, D)** in the shoot of common bean plants cultivated without water deficit (WWD) [80% of the water retention capacity (WRC)], with deficit moderate water deficit (MWD) (60% of WRC), and with severe water deficit (SWD) (40% of WRC), combined with doses of Si supplied via fertigation: 0 kg/ha, 4 kg/ha, 8 kg/ha, and 12 kg/ha, without (K0) and with (K1) potassium fertilization. Letters show differences for water regimes (WR) at each dose of Si (*p* < 0.05, Tukey’s test). * and ** indicate significance at 1% and 5% probability, respectively, and ns indicates non-significance by the F test.

Silicon accumulation in the shoot of the plants without K fertilization showed an effect of the interaction between the factors (WR × Si) (*p* < 0.01) (**Figure 2A**). In plants with K fertilization, there was only an effect of the water regime and Si doses isolated factors (both at *p* < 0.01) (**Figure 2B**). The Si doses provided an effect with quadratic polynomial regression adjustment in the three water regimes, regardless of the potassium condition.

Plants without potassium fertilization obtained their maximum accumulations of Si at 68.7 mg, 38.2 mg, and 21.4 mg per plant at doses of 6.8 kg/ha, 6.7 kg/ha, and 6.5 kg/ha of Si, respectively, for WWD, MWD, and SWD (**Figure 2A**). Plants with potassium fertilization showed the maximum accumulation of Si at 59.8 mg, 37.8 mg, and 21.1 mg per plant, obtained at doses of 5.8 kg/ha, 6.2 kg/ha, and 7.0 kg/ha, respectively (**Figure 2B**). The lowest Si accumulation was observed under MWD and SWD at all Si doses compared to WWD regardless of the K condition. The lowest Si accumulations were observed in plants under MWD and SWD at all Si doses evaluated and under the two conditions of potassium fertilization.

**Figure 3:**
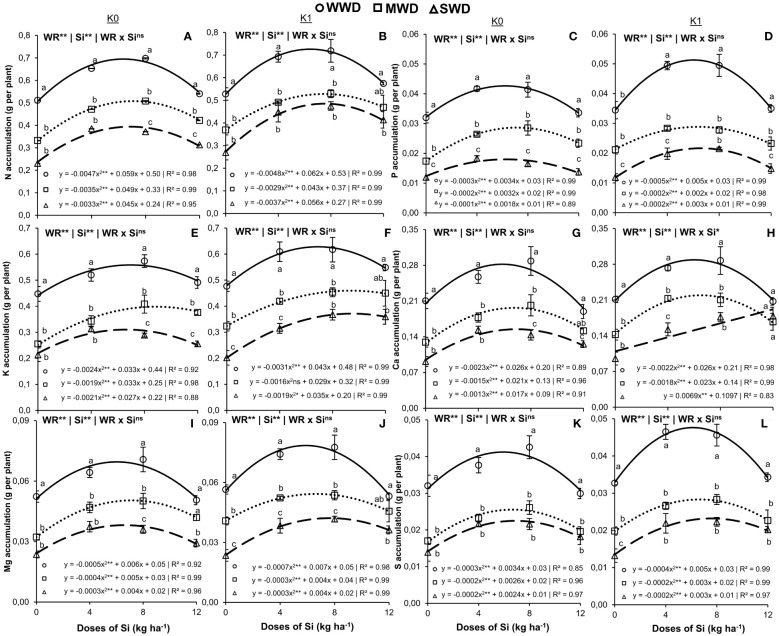
Accumulation of nitrogen (N) **(A, B)**, phosphor (P) **(C, D)**, potassium (K) **(E, F)**, calcium (Ca) **(G, H)**, magnesium (Mg) **(I, J)**, and sulfur (S) **(K, L)** in the shoot of common bean plants cultivated without water deficit (WWD) [80% of the water retention capacity (WRC)], with deficit moderate water deficit (MWD) (60% of WRC), and with severe water deficit (SWD) (40% of WRC), combined with doses of Si supplied via fertigation: 0 kg/ha, 4 kg/ha, 8 kg/ha, and 12 kg/ha, without (K0) and with (K1) potassium fertilization. Letters show differences for water regimes (WR) at each dose of Si (*p* < 0.05, Tukey’s test). * and ** indicate significance at 1% and 5% probability, respectively, and ns indicates non-significance by the F test.

Accumulations of C (**Figures 2C, D**) and N and P (**Figures 3A–D**) in the shoot of common bean plants were influenced by the isolated effects of the water regime and Si doses (all with *p* < 0.01) regardless of potassium fertilization. The Si doses caused an effect with quadratic polynomial adjustment for all water regimes in the cultivation without and with potassium fertilization. The maximum accumulations of C in the plants that received potassium fertilization were 10.9 g, 8.6 g, and 7.0 g per plant at doses of 6.0 kg/ha, 7.2 kg/ha, and 8.0 kg/ha of Si, respectively, for WWD, MWD, and SWD. When potassium fertilization was not performed, the highest C accumulations were 9.8 g, 8.0 g, and 6.4 g per plant at doses of 5.6 kg/ha, 7.3 kg/ha, and 7.0 kg/ha, respectively. Plants cultivated without potassium fertilization and water deficit showed the highest accumulations of C in all doses of Si compared to MWD and SWD. This fact also occurred when the plants received fertilization with K, except for the highest dose of Si (12 kg/ha) (**Figures 2C, D**).

The highest accumulations of N in shoots were 0.7 g, 0.5 g, and 0.4 g per plant obtained at doses of 6.3 kg/ha, 7.0 kg/ha, and 7.0 kg/ha of Si, respectively, for WWD, MWD, and SWD, in plants without K (**Figure 3A**). Under the K condition, the maximum N accumulations were 0.7 g, 0.5 g, and 0.5 g per plant obtained at doses of 6.5 kg/ha, 7.4 kg/ha, and 7.6 kg/ha of Si, respectively, for WWD, MWD, and SWD (**Figure 3B**). Plants under WWD under both K conditions had the highest N accumulations at all Si doses tested compared to MWD and SWD plants, except for the highest Si dose in plants with potassium fertilization.

The maximum accumulations of P were 0.04 g, 0.03 g, and 0.02 g per plant at doses of 5.7 kg/ha, 8.0 kg/ha, and 9.0 kg/ha, respectively, for WWD, MWD, and SWD and without potassium fertilization (**Figure 3C**). Under the potassium fertilization condition, the maximum accumulations of P were 0.04 g, 0.03 g, and 0.02 g per plant, obtained at doses of 5.0 kg/ha, 5.0 kg/ha, and 7.5 kg/ha of Si, respectively, for WWD, MWD, and SWD (**Figure 3D**). Phosphorus accumulation was higher in WWD plants under the two K conditions at all Si doses tested compared to that in MWD and SWD plants.

The water regime and Si doses isolated factors (both at *p* < 0.01) influenced K accumulation in the shoot of the plants under both potassium fertilization conditions. The Si doses caused an effect with quadratic polynomial adjustment for all water regimes (**Figures 3E, F**) in the cultivation without and with potassium fertilization. Plants without potassium fertilization showed maximum K accumulation of 0.6 g, 0.4 g, and 0.3 g per plant at doses of 6.9 kg/ha, 8.7 kg/ha, and 6.4 kg/ha, respectively, for WWD, MWD, and SWD (**Figure 3E**). The maximum accumulations of K in the plants that received potassium fertilization were 0.6 g, 0.5 g, and 0.4 g per plant at doses of 6.9 kg/ha, 9.0 kg/ha, and 9.2 kg/ha of Si, respectively, for WWD, MWD, and SWD (**Figure 3F**). Plants cultivated without potassium fertilization and water deficit had the highest K accumulations in all Si doses compared to plants under MWD and SWD. This fact also occurred when the plants received fertilization with K, except for the highest dose of Si (12 kg/ha).

The accumulations of Ca, Mg, and S in the shoot of bean plants were influenced by the isolated effects of the water regime and Si dose factors (all with *p* < 0.01) regardless of potassium fertilization (**Figures 3G–L**), except for Ca accumulation in plants with K fertilization, which had an interaction effect (WR × Si) (*p* < 0.05) (**Figure 3H**). The Si doses tested provided a quadratic polynomial regression adjustment for the accumulations of Ca, Mg, and S in the three water regimes and under both conditions of K fertilization, except for the accumulation of Ca in plants under SWD and with potassium fertilization, where there was a linear fit.

Plants that did not receive K fertilization showed a maximum accumulation of Ca of 0.28 g, 0.20 g, and 0.15 g per plant with doses of 5.8 kg/ha, 6.9 kg/ha, and 6.5 kg/ha of Si, respectively, for WWD, MWD, and SWD (**Figure 3G**). Plants that received potassium fertilization showed maximum Ca accumulation of 0.29 g and 0.21 g per plant at doses of 5.9 kg/ha and 6.4 kg/ha of Si, respectively, for WWD and MWD. The increased rate of Ca accumulation in plants cultivated with potassium fertilization and under SWD was 0.0069 g per plant for every 1 kg/ha of Si (**Figure 3H**). Calcium accumulation was higher under the two K conditions in the WWD compared to SWD plants, except for the 12 kg/ha dose in the plants with potassium fertilization, in which the Ca accumulation did not differ between the water regimes.

The water conditions of WWD, MWD, and SWD provided maximum Mg accumulations of 0.069 g, 0.046 g, and 0.035 g per plant at doses of 6.1 g/kg, 6.4 g/kg, and 7.2 g/kg of Si in the cultivation without potassium fertilization, and 0.068 g, 0.053 g, and 0.033 g per plant at doses of 5.0 g/kg, 6.7 g/kg, and 6.7 g/kg of Si in the cultivation with potassium fertilization, respectively (**Figures 3I, J**). Magnesium accumulation in plants with and without K fertilization was lower in plants under SWD at all Si doses tested. However, MWD plants at the dose of 12 kg/ha of Si did not differ in Mg accumulation from WWD plants under the two K conditions.

The maximum accumulations of S in plants that did not receive K fertilization were 0.04 g, 0.03 g, and 0.02 g per plant with doses of 5.7 kg/ha, 6.5 kg/ha, and 6.0 kg/ha of Si, respectively, for WWD, MWD, and SWD (**Figure 3K**). However, in plants that received potassium fertilization, the maximum accumulations of S were 0.05 g, 0.03 g, and 0.02 g per plant at doses of 6.2 kg/ha, 7.5 kg/ha, and 7.5 kg/ha of Si, respectively, for WWD, MWD, and SWD (**Figure 3L**). At all evaluated Si doses, S accumulation was greater in plants grown without water deficit, under both K conditions, compared to plants under water deficit, whether moderate or severe.

**Figure 4:**
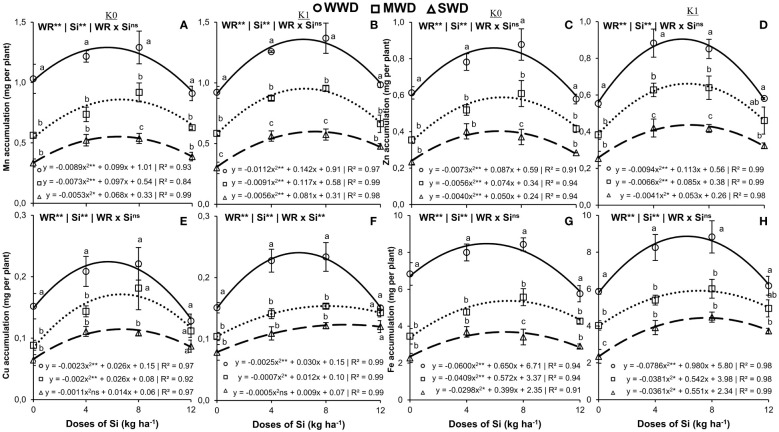
Accumulation of manganese (Mn) (N) **(A, B)**, zinc (Zn) **(C, D)**, copper (Cu) **(E, F)**, and iron (Fe) **(G, H)** in the shoot of common bean plants cultivated without water deficit (WWD) [80% of the water retention capacity (WRC)], with deficit moderate water deficit (MWD) (60% of WRC), and with severe water deficit (SWD) (40% of WRC), combined with doses of Si supplied via fertigation: 0 kg/ha, 4 kg/ha, 8 kg/ha, and 12 kg/ha, without (K0) and with (K1) potassium fertilization. Letters show differences for water regimes (WR) at each dose of Si (*p* < 0.05, Tukey’s test). * and ** indicate significance at 1% and 5% probability, respectively, and ns indicates non-significance by the F test.

The water regime and Si doses isolated factors (both at *p* < 0.01) favored the accumulation of Mn and Zn in the shoot under both potassium fertilization conditions (**Figures 4A–D**). Furthermore, they favored the accumulation of Cu in plants without potassium fertilization (both at *p* < 0.01) (**Figure 4E**). The effect of the interaction (WR × Si) was significant only for the accumulation of Cu in the shoot of plants cultivated with potassium fertilization (*p* < 0.01) (**Figure 4F**). The accumulations of Mn, Zn, and Cu in plants without potassium fertilization and with potassium fertilization showed a quadratic polynomial fit with the Si doses applied (**Figures 4A–F**).

The maximum values in the shoot of plants without K were 1.3 mg, 0.9 mg, and 0.5 mg per plant for Mn accumulation at doses of 5.6 kg/ha, 6.6 kg/ha, and 6.4 kg/ha of Si; 0.8 mg, 0.6 mg, and 0.4 mg per plant for Zn accumulation at doses of 6.0 kg/ha, 6.6 kg/ha, and 6.2 kg/ha of Si; and 0.2 mg, 0.2 mg, and 0.1 mg per plant for Cu accumulation at doses of 5.6 kg/ha, 6.5 kg/ha, and 6.4 kg/ha of Si, respectively, for WWD, MWD, and SWD. The accumulations of Mn and Zn in plants without K fertilization were lower in the water deficit regimes in all Si doses compared to the plants under adequate water conditions. However, Cu accumulation at doses of 8 kg/ha and 12 kg/ha of Si in MWD plants did not differ from plants under WWD (**Figures 4A, C, E**).

In plants with potassium fertilization, the maximum values were 1.4 mg, 1.0 mg, and 0.6 mg per plant for Mn accumulation at doses of 6.3 kg/ha, 6.4 kg/ha, and 7.2 kg/ha of Si; 0.9 mg, 0.6 mg, and 0.4 mg per plant for Zn accumulation at doses of 6.0 kg/ha, 6.4 kg/ha, and 6.5 kg/ha of Si; and 0.24 mg, 0.15 mg, and 0.11 mg per plant for Cu accumulation at doses of 6.0 kg/ha, 8.6 kg/ha, and 9.0 kg/ha of Si, respectively, for WWD, MWD, and SWD. The accumulation of Mn, Zn, and Cu in plants fertilized with K was higher under adequate water conditions, except for the Si dose of 12 kg/ha, where there was no difference in water regimes for Cu accumulation (**Figures 4B, D, F**).

The water regime and Si doses isolated factors (both at *p* < 0.01) influenced the accumulation of Fe in shoots under the two potassium fertilization conditions (**Figures 4G, H**). The Si doses provided an effect with quadratic polynomial regression adjustment in the three water regimes, regardless of the potassium condition. Plants without potassium fertilization obtained their maximum accumulations of 8.5 mg, 5.4 mg, and 3.7 mg per plant of Fe at doses of 5.4 kg/ha, 7.0 kg/ha, and 6.7 kg/ha of Si, respectively, for WWD, MWD, and SWD (**Figure 4G**). Plants with potassium fertilization showed the maximum accumulation of 8.9 mg, 5.9 mg, and 4.4 mg per plant at doses of 6.2 kg/ha, 7.1 kg/ha, and 7.6 kg/ha of Si, respectively, for WWD, MWD, and SWD (**Figure 4H**).

Iron (Fe) accumulation in plants not fertilized and fertilized with K in water deficit (MWD and SWD) was lower at doses of 0 kg/ha, 4 kg/ha, and 8 kg/ha of Si compared to plants under optimal water conditions. However, MWD plants that were not fertilized with K but fertilized with 8 kg/ha of Si still showed higher Fe accumulation than plants under SWD. In plants fertilized with K, however, this response occurred only in plants without Si (0 kg/ha).

**Figure 5:**
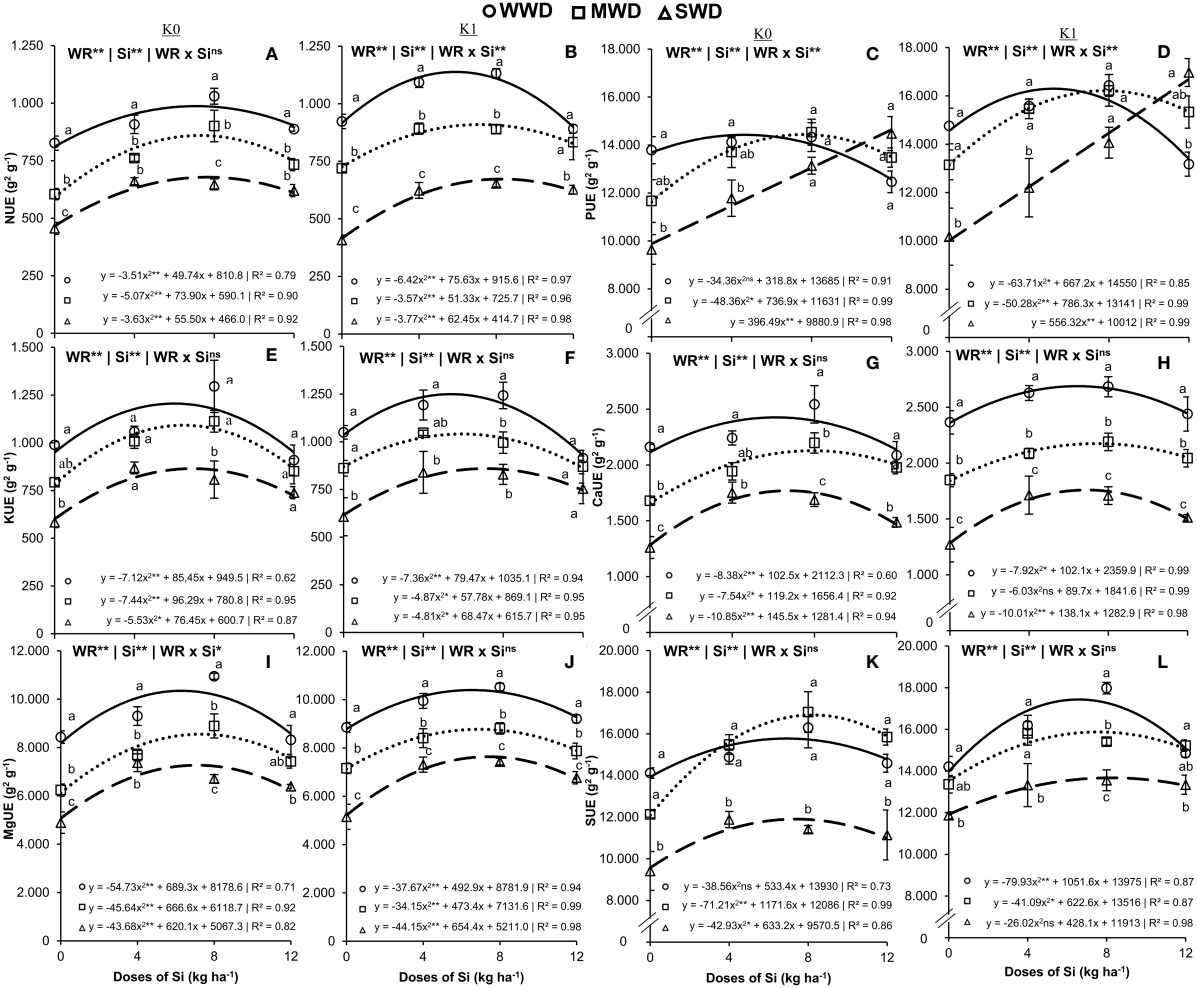
Efficiency of use the nitrogen (NUE) **(A, B)**, phosphorus (PUE) **(C, D)**, potassium (KUE) **(E, F)**, calcium (CaUE) **(G, H)**, magnesium (MgUE) **(I, J)**, and sulfur (SUE) **(K, L)** in the common bean plants cultivated without water deficit (WWD) [80% of the water retention capacity (WRC)], with deficit moderate water deficit (MWD) (60% of WRC), and with severe water deficit (SWD) (40% of WRC), combined with doses of Si supplied via fertigation: 0 kg/ha, 4 kg/ha, 8 kg/ha, and 12 kg/ha, without (K0) and with (K1) potassium fertilization. Letters show differences for water regimes (WR) at each dose of Si (*p* < 0.05, Tukey’s test). * and ** indicate significance at 1% and 5% probability, respectively, and ns indicates non-significance by the F test.

The use efficiency of N had an effect on isolated factors in plants that did not receive K application (**Figure 5A**) and on the interaction (Si × WD) when potassium fertilization was performed (**Figure 5B**). Under the two potassium fertilization conditions, the Si doses provided an effect with quadratic polynomial adjustment in the three water regimes evaluated. The maximum N use efficiencies in plants cultivated under WWD were 987.1 g^2^/g and 1,138.4 g^2^/g at Si doses of 7.1 kg/ha and 5.9 kg/ha for cultivation without and with potassium fertilization, respectively. Under MWD, the maximum efficiencies were 859.6 g^2^/g and 909.9 g^2^/g using Si doses of 7.3 kg/ha and 7.2 kg/ha for cultivation without and with potassium fertilization, respectively. Regarding the SWD, 678.3 g^2^/g and 678.2 g^2^/g were obtained when applying Si doses of 7.6 kg/ha and 8.3 kg/ha via fertigation, respectively, for cultivation without and with potassium fertilization (**Figures 5A, B**). The adequate water regime provided higher N use efficiencies regarding MWD and SWD in all Si doses, in plants without potassium fertilization, and only in Si doses of 0 kg/ha, 4 kg/ha, and 8 kg/ha in plants with fertilization with K.

The use efficiency of K had an isolated effect on the factors water regime and Si doses (both at *p* < 0.01) in plants with and without potassium fertilization (**Figures 5E, F**). The impacts provided by the Si doses had a quadratic polynomial fit. The maximum K use efficiencies were 1,205.7 g^2^/g, 1,092.1 g^2^/g, and 867.9 g^2^/g at Si doses of 6.0 kg/ha, 6.5 kg/ha, and 6.9 kg/ha, respectively, for WWD, MWD, and SWD in plants that did not receive potassium fertilization (**Figure 5E**). In plants fertilized with K, the maximum K efficiencies obtained were 1,249.5 g^2^/g, 1,040.6 g^2^/g, and 859.4 g^2^/g at doses of 5.4 kg/ha, 5.9 kg/ha, and 7.1 kg/ha of Si, respectively, for WWD, MWD, and SWD (**Figure 5F**). Without potassium fertilization, plants under WWD showed higher K use efficiencies at doses of 0 kg/ha and 8 kg/ha of Si compared to plants under SWD. However, this scenario occurred with potassium fertilization at doses of 0 kg/ha, 4 kg/ha, and 8 kg/ha of Si.

The interaction effect (WR × Si) influenced the P use efficiency (*p* < 0.01) under the two K conditions (**Figures 5C, D**) and the Mg use efficiency (*p* < 0. 01) in plants that did not receive potassium fertilization (**Figure 5I**). Only the water regime and Si doses isolated factors were significant (both at *p* < 0.001) for Ca efficiency under both K fertilization conditions (**Figures 5G, H**) and for Mg efficiency with potassium fertilization (**Figure 5J**). The Si doses provided a quadratic polynomial regression fit for the P, Ca, and Mg use efficiencies in the three water regimes and under both K fertilization conditions, except for the P use efficiency in plants under SWD in the two conditions of fertilization with K, in which there was a linear adjustment.

The maximum values of P use efficiency in plants cultivated under WWD were 14,424.5 g^2^/g and 16,296.9 g^2^/g at Si doses of 4.6 kg/ha and 5.2 kg/ha for cultivation without and with potassium fertilization, respectively. Under MWD, the maximum P use efficiencies were 14,438.4 g^2^/g and 16,215.0 g^2^/g using Si doses of 7.6 kg/ha and 7.8 kg/ha for cultivation without and with potassium fertilization, respectively. Regarding the SWD regime, the increase rates were 396.5 g^2^/g and 5.56.3 g^2^/g for each 1 kg/ha of Si applied via fertigation, respectively, for cultivation without and with potassium fertilization (**Figures 5C, D**). There was no difference in P use efficiency between regimes at higher Si doses (8 kg/ha and 12 kg/ha) in plants without K fertilization. With potassium fertilization, plants under SWD showed greater P use efficiency than WWD plants at the 12 kg/ha dose. In the absence of Si or low dose (0 kg/ha and 4 kg/ha Si), plants under SWD showed lower P use efficiencies under both K conditions.

We showed that in the plants without potassium fertilization, the Ca use efficiencies were 2,420.8 g^2^/g, 2,127.3 g^2^/g, and 1,769.4 g^2^/g at Si doses of 6.12 kg/ha, 7.9 kg/ha, and 6.71 kg/ha, respectively, for WWD, MWD, and SWD. Under the K fertilization condition, the maximum Ca use efficiencies were 2,689.0 g^2^/g, 2,175.0 g^2^/g, and 1,759.5 g^2^/g at Si doses of 6.5 kg/ha, 7.4 kg/ha, and 6.9 kg/ha, respectively, for WWD, MWD, and SWD (**Figures 5G, H**). Calcium (Ca) use efficiency in plants under SWD was lower in all Si doses and under both K conditions compared to plants under WWD. However, at Si doses of 4 kg/ha and 12 kg/ha, plants without K fertilization under MWD did not differ from plants under optimal water conditions.

The maximum values of Mg use efficiency in plants grown with and without potassium fertilization and under WWD were 10,349.2 g^2^/g and 10,394.7 g^2^/g at Si doses of 6.3 kg/ha and 6.5 kg/ha, respectively. In the MWD regime, the maximum Mg use efficiencies were 8,553.0 g^2^/g and 8,772.4 g^2^/g at Si doses of 7.3 kg/ha and 6.9 kg/ha, respectively, for cultivation without and with potassium fertilization. In the SWD regime, 7,268.4 g^2^/g and 7,636.0 g^2^/g were obtained at Si doses of 7.1 kg/ha and 7.4 kg/ha, respectively, for cultivation without and with potassium fertilization (**Figures 5I, J**). Magnesium (Mg) use efficiencies were lower in unfertilized and K-fertilized plants under MWD and SWD at all Si doses compared to plants under WWD. However, at the dose of 12 kg/ha of Si for the MWD regime in plants without K fertilization, the Mg use efficiency did not differ between the WWD and SWD regimes.

The isolated factors WR and Si influenced S use efficiency (both *p* < 0.01) in both K (**Figures 5K, L**). The Si doses provided a quadratic polynomial regression in the three water regimes and under both K fertilization conditions. The maximum S use efficiencies were 15,774.6 g^2^/g, 16,905.0 g^2^/g, and 11,905.7 g^2^/g at Si doses of 6.9 kg/ha, 8.2 kg/ha, and 7.4 kg/ha, respectively, for WWD, MWD, and SWD in plants without potassium fertilization (**Figure 5K**). In the plants fertilized with K, the maximum S efficiencies obtained were 17,433.8 g^2^/g, 15,874.4 g^2^/g, and 13,674.0 g^2^/g at Si doses of 6.6 kg/ha, 7.6 kg/ha, and 8.2 kg/ha, respectively, for WWD, MWD, and SWD (**Figure 5L**). Under both potassium conditions, the plants under WWD and MWD showed higher S use efficiencies at all the Si doses evaluated compared to the plants under SWD, except for the 8 kg/ha Si dose in the presence of K fertilization.

**Figure 6:**
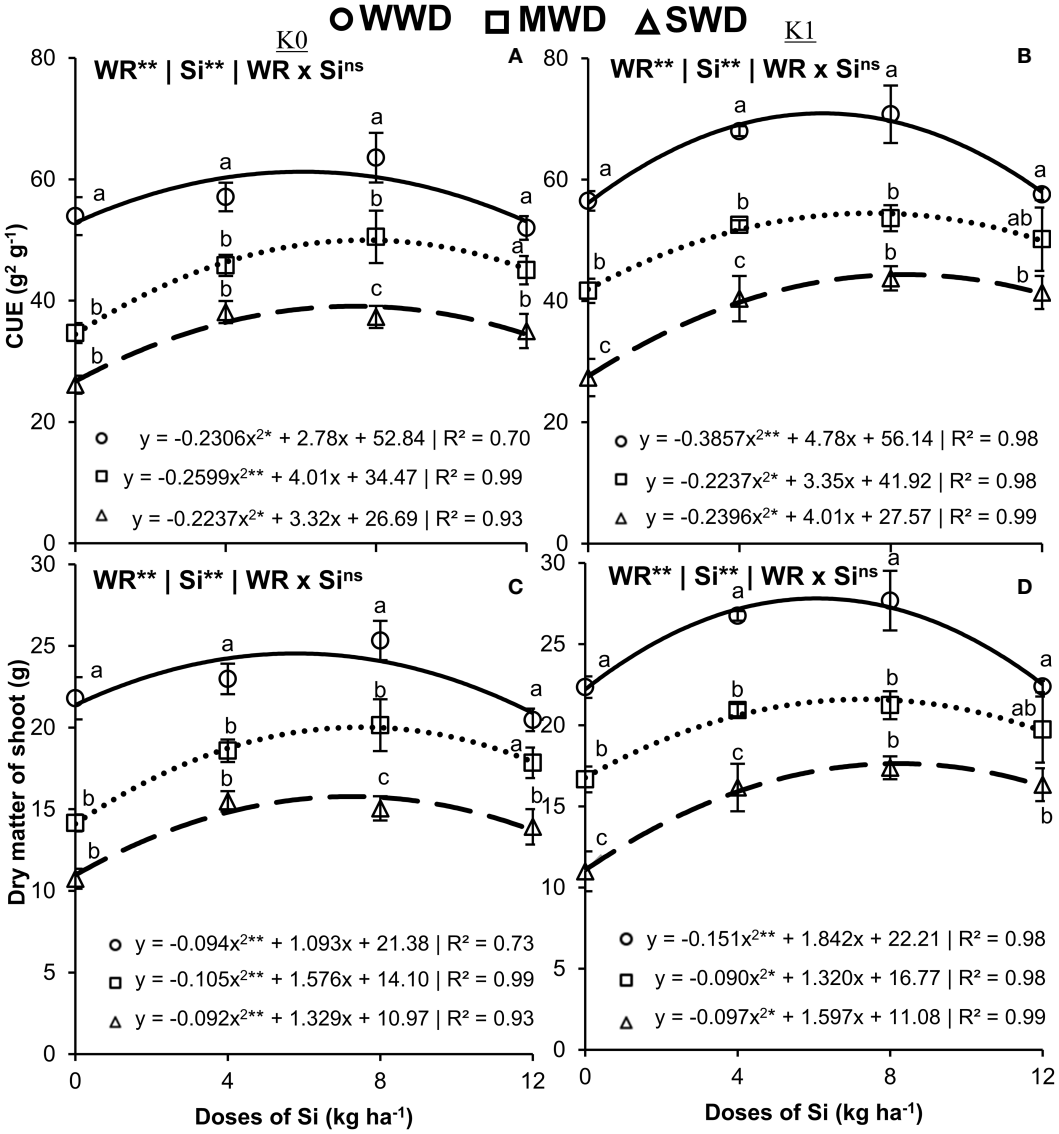
Efficiency of use of carbon (CUE) **(A, B)** and dry matter of shoot **(C, D)** in the common bean plants cultivated without water deficit (WWD) [80% of the water retention capacity (WRC)], with deficit moderate water deficit (MWD) (60% of WRC) and with severe water deficit (SWD) (40% of WRC), combined with doses of Si supplied via fertigation: 0 kg/ha, 4 kg/ha, 8 kg/ha, and 12 kg/ha, without (K0) and with (K1) potassium fertilization. Letters show differences for water regimes (WR) at each dose of Si (*p* < 0.05, Tukey’s test). * and ** indicate significance at 1% and 5% probability, respectively, and ns indicates non-significance by the F test.

The efficiency of C use was influenced by the isolated effect of the factors (both with *p* < 0.01) under both potassium fertilization conditions, with a quadratic polynomial adjustment for the effect of Si doses under the three water conditions (**Figures 6A, B**). With potassium fertilization, the highest C use efficiencies were 61.2 g^2^/g, 49.9 g^2^/g, and 39 g^2^/g obtained with the supply of Si doses of 6.0 kg/ha, 7.7 kg/ha, and 7.4 kg/ha in the absence of potassium fertilization and 70.9 g^2^/g, 54.5 g^2^/g, and 44.3 g^2^/g in the Si doses of 6.2 kg/ha, 7.5 kg/ha, and 8.4 kg/ha (**Figures 6A, B**). The C use efficiencies were higher in unfertilized and K-fertilized plants under MWD that received Si doses of 0 kg/ha, 4 kg/ha, and 8 kg/ha than in plants under WWD and SWD. Supplying Si at a dose of 12 kg/ha decreased C efficiency, which was similar in plants under WWD and MWD.

**Figure 7:**
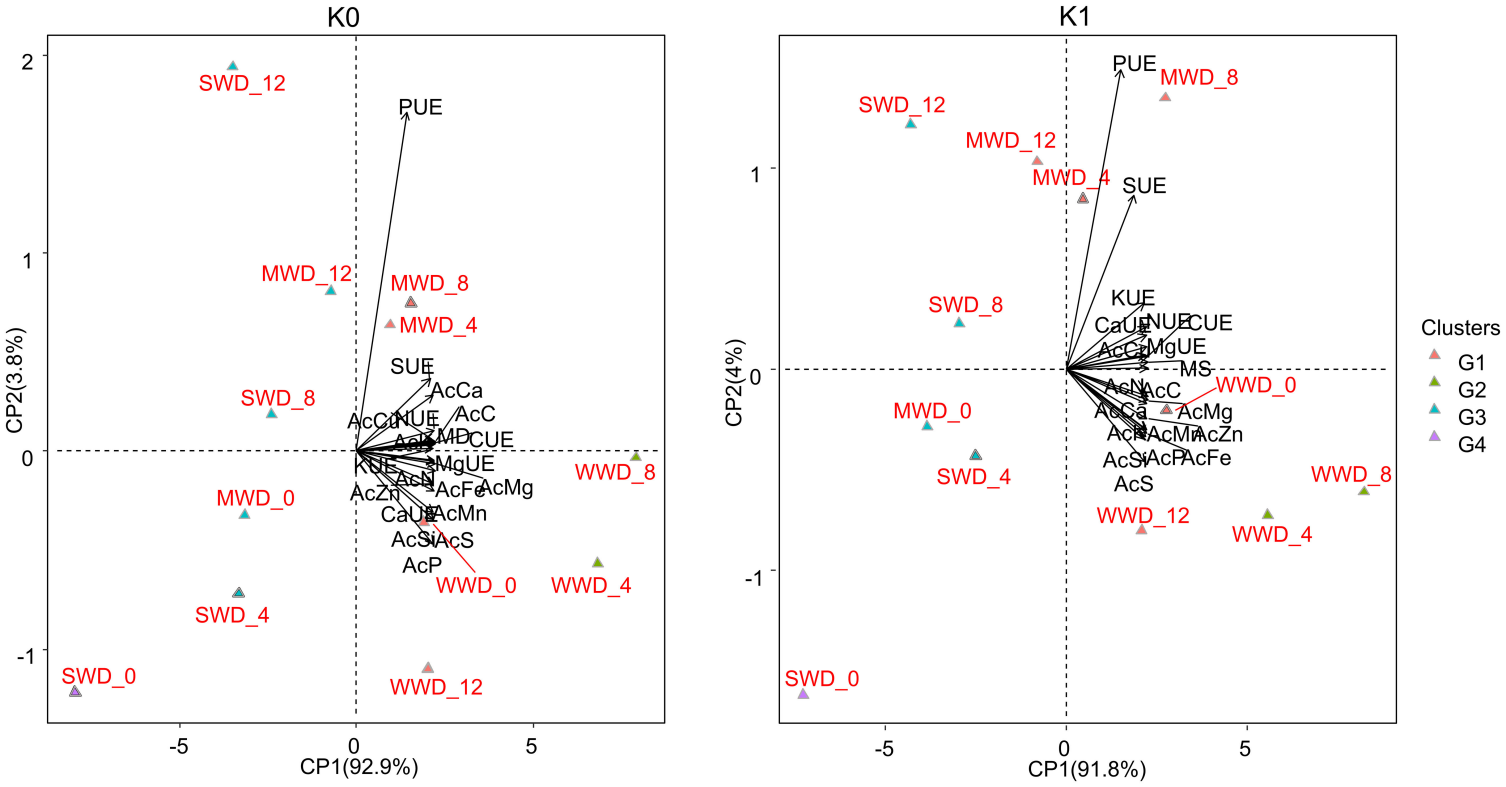
Principal component analysis of accumulation variables (Ac) of C, N, P, K, Ca, Mg, S, Mn, Cu, Fe and Zn; use efficiencies (UE) of C, N, P, K, Ca, Mg, and S; and dry matter production (DM) of common bean plants cultivated without water deficit (WWD) [80% of the water retention capacity (WRC)], with moderate water deficit (MWD) (60% of WRC), and with severe water deficit (SWD) (40% of WRC), combined with doses of Si supplied via fertigation: 0 kg/ha, 4 kg/ha, 8 kg/ha, and 12 kg/ha, without (K0) and with (K1) potassium fertilization. G1, G2, G3, and G4 refer to grouping treatments into hierarchical groups using Euclidean distance.

The maximum dry mass values of the aerial part were obtained in plants without potassium fertilization (24.5 g, 20.01 g, and 15.78 g) and with potassium fertilization (27.81 g, 21.59 g, and 17.63 g) at Si doses of 5.78 kg/ha, 7.50 kg/ha, and 7.23 kg/ha and 6.08 kg/ha, 7.31 kg/ha, and 8.21 kg/ha, respectively, for the WWD, MWD, and SWD regimes (**Figures 6C, D**). At a dose of 12 kg/ha, plants grown under MWD did not differ from plants grown under WWD under the condition without potassium fertilization. Plants grown under SWD with a supply of 4 kg/ha of Si had a dry mass similar to that of plants grown under MWD (**Figure 6C**). However, plants fertilized with K and under SWD with higher doses of Si (8 kg/ha and 12 kg/ha) had the same dry mass as plants under MWD (**Figure 6D**). PCA explained 92.9% and 3.8%, and 91.8% and 4% in plants grown without and with potassium fertilization, respectively (**Figure 7**).

In bean cultivation without potassium fertilization, the accumulation (Ac) of Si, N, P, Mg, S, Mn, Fe, and Zn and the use efficiencies (UE) of Ca, Mg, and K were associated with the WWD regime at doses of 0 kg/ha and 4 kg/ha of Si. The accumulation of C, Ca, and Cu and the use efficiency of C, N, and S were associated with the WWD regime at a Si dose of 8 kg/ha and the MWD regime at Si doses of 4 kg/ha and 8 kg/ha. In the cultivation scenario with potassium fertilization, the accumulation (Ac) of C, the UE of C, N, K, Ca, and Mg, and dry mass production were associated with the WWD regime at a dose of 0 kg/ha of Si but also with the WWD regime at doses of 4 kg/ha and 8 kg/ha of Si.

Cluster analysis showed that applying intermediate doses of Si (4 kg/ha and 8 kg/ha) under adequate water conditions is more efficient, regardless of potassium fertilization. Similarly, doses of 4 kg/ha and 8 kg/ha of Si efficiently improved the growth of plants under moderate water deficit, grouping them with plants under WWD that received Si doses of 0 kg/ha and 12 kg/ha in the absence of potassium fertilization. However, with potassium fertilization, Si use efficiency was improved, and the 12 kg/ha dose of Si under MWD was included in this group. Moreover, plants under SWD and without any Si supply were individually separated under both potassium conditions, demonstrating that water deficiency without Si causes the greatest damage to the development of bean plants in the field (**Figure 7**).

**Figure 8:**
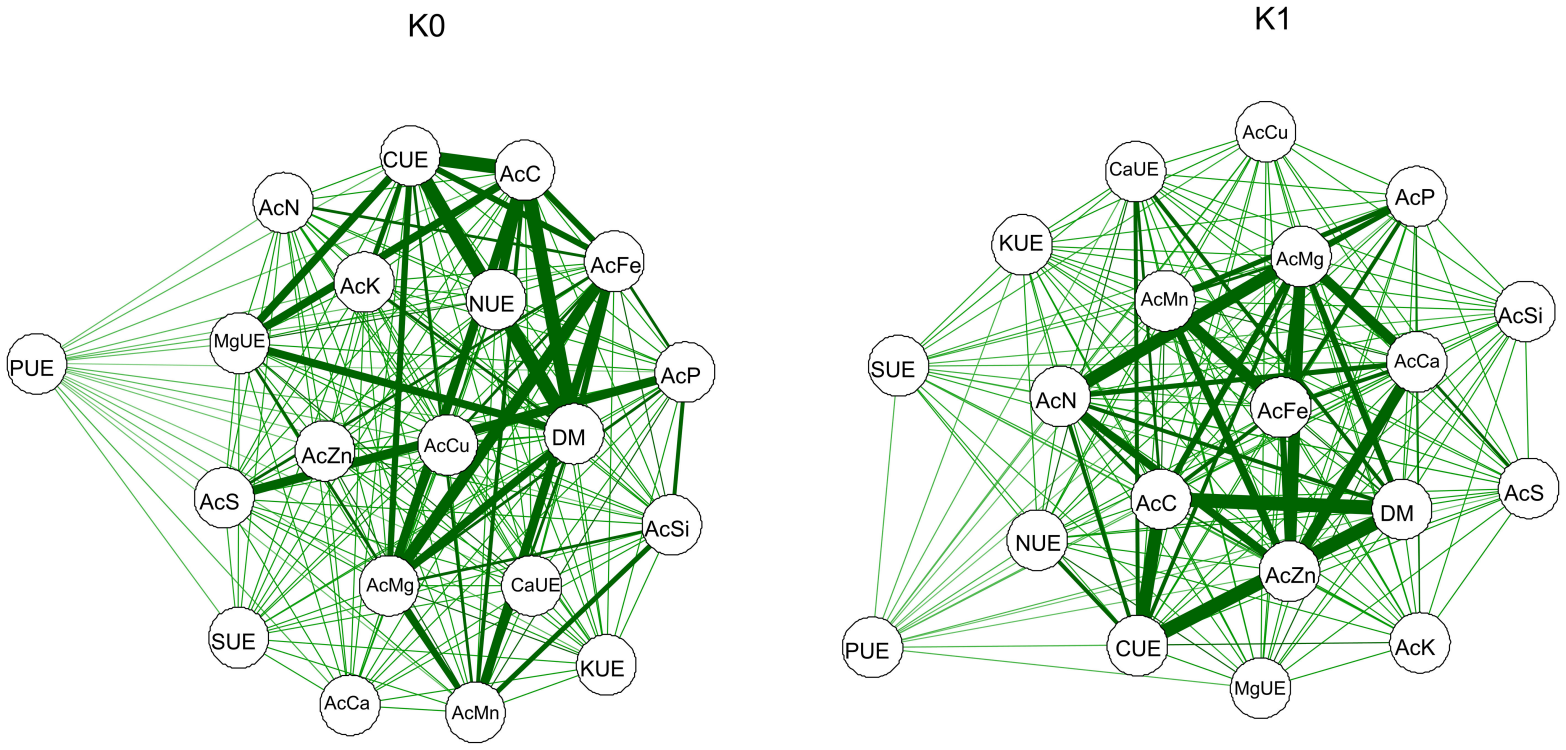
Pearson’s correlation network between the variable accumulation (Ac) of C, N, P, K, Ca, Mg, S, Mn, Cu, Fe, and Zn; use efficiencies (EU) of C, N, P, K, Ca, Mg, and S; and dry matter production (DM) in common bean plants cultivated without water deficit (WWD) [80% of the water retention capacity (WRC)], with moderate water deficit (MWD) (60% of WRC), and with severe water deficit (SWD) (40% of WRC), combined with doses of Si supplied via fertigation: 0 kg/ha, 4 kg/ha, 8 kg/ha, and 12 kg/ha, without (K0) and with (K1) potassium fertilization. Positive correlations are highlighted in green, and the stroke thickness indicates the strength of the correlation.

The results revealed a high Pearson’s correlation between the variables used in the network. In plants grown without potassium fertilization, it was evident that dry mass production (DM) had a strong correlation with accumulation (AcC), C use efficiency (CUE), and N use efficiency (NUE). The accumulation of Fe (AcFe) and Mg (AcMg) and the use efficiencies of Ca (CaUE) and Mg (MgUE) were also positively correlated with DM, however, to a lesser extent than the previous variables. When potassium was applied, AcC, CUE, and DM had a similar positive correlation. Furthermore, DM was also strongly influenced by Zn accumulation (AcZn). Positive correlations of a lesser degree of intensity were also observed for the accumulation of N (AcN) and Mg (AcMg) and with Ca use efficiency (CaUE). It is worth noting that P use efficiency (PUE) showed a low correlation with DM under both potassium fertilization conditions (**Figure 8**).

## 4 Discussion

The decrease in soil water content intensified by climate change has caused physiological, biochemical, and morphological damage in several species (Besharat et al., 2020). It includes secondary effects that are still little reported, such as nutritional deficiency, which is more severe in the common bean, a species sensitive to drought.

Recent research reported that the application of Si can improve nutrient absorption (Buchelt et al., 2020; Araújo et al., 2022; Teixeira et al., 2022a). However, studies have been limited to nutrient-deficient growing conditions (Etesami and Jeong, 2018) and do not address soils with excellent fertility that occur in technified, irrigated crops, which is this study’s case. Moreover, studies are predominantly restricted to controlled environment trials with plants grown in pots, and the results do not apply to field crops. Therefore, it became necessary to conduct field studies aimed at improving the application of Si through fertigation to promote the absorption of this element by the plant.

Our study achieved this objective. Its results showed that Si accumulation in the shoot of the legume had an agronomically significant increase with the application of the element through fertigation in bean plants cultivated under all water conditions studied, without and with potassium fertilization. This fact demonstrates that even in plants that are not considered accumulators of the beneficial element, enriching leaf tissues with Si through the efficient application of the element through fertigation with an adequate dose is possible. The fertigation efficiency was the result of fluid application using a soluble source stabilized with sorbitol, which favors the predominance of monomeric forms of Si in solution, reducing the rates of Si polymerization in the soil (Birchall, 1995) and increasing absorption by plants (Teixeira et al., 2022a).

The decrease in polymerization was also favored using Si concentrations below the critical polymerization range (3.5 mmol/L) (Birchall, 1995), allowing the use of fewer doses in the field than 20 kg/ha. The most suitable dose of Si to be applied that generated the highest content of this element in the plant increased with the severity of the water deficit, with the recommended doses of 6 kg/ha, 7 kg/ha, and 8 kg/ha of Si for the adequate water condition of cultivation, moderate water deficit, and severe water deficit, respectively. This result allows us to accept hypothesis i) that there is an optimum Si dose for fertigation for its use in irrigated bean crops, which is affected by the different water levels in the soil. The use of these doses highlights the main novelty of this research, which is the efficient application of Si in irrigated crops, using doses that correspond to 3.0%, 3.5%, and 4.0% of the dose used in other experiments that applied calcium silicate to the soil (dose equivalent to 200 kg/ha) (Amin et al., 2014; 2018). The decrease in the dose applied through fertigation had already been reported in a previous experiment, in which the authors demonstrated that using an 11% lower dose already promoted an important effect in mitigating the water deficit in corn plants grown in pots (Teixeira et al., 2022a).

The high viability of Si fertigation to promote optimal Si absorption in a leguminous plant proven here is a very relevant finding, as it increases the expectation of this element being a possible modulator of mechanisms to mitigate water stress, which is this study’s objective.

Therefore, the effect of Si as an inducer of nutritional defense mechanisms in bean plants under field conditions is a novelty, as current research has shown effects only on growth and production, especially in accumulator plants grown in controlled environments (Teixeira et al., 2022b). However, plants with a low capacity to accumulate the element, such as common beans, maintain the highest Si content in the root. Regarding the shoot, the beneficial effects are restricted to physiological mechanisms (dos Santos Sarah et al., 2021), without clarifying the nutritional mechanisms involved.

The role of silicon in plants is crucial in preventing damage to the xylem vessels and regulating stomatal opening under drought conditions. It is essential to ensure the efficient transportation of water and nutrients throughout the plant during water scarcity (Sapre and Vakharia, 2016). The preservation of xylem vessels is due to the formation of phytoliths and polymerization of insoluble Si in the cell walls and around the vascular bundle, reducing water loss and controlling transpiration (Mecfel et al., 2007), and it can favor the absorption of nutrients by the plants.

Thus, to discover which nutrients are most involved in the action of Si as a nutritional modulating agent, the nutritional accumulations and efficiency of each nutrient must be evaluated. The accumulation of N and P was affected by the water deficit and the application of Si, with an effect on the dry matter production because these nutrients are closely related to the photosynthetic capacity (Crous et al., 2017), which also depends on the water status. A large portion of accumulated N is invested in the photosynthetic machinery, so plants with higher N use efficiency can improve Rubisco carboxylation and electron transport rates (Guan and Wen, 2011). Thus, bean plants under moderate and severe water deficits, when receiving Si, do not have limitations in N absorption. They improve photosynthetic performance and then show dry matter gain.

Similar results were obtained in sugarcane plants under water deficit, with effects attributed to increased levels of chlorophyll and photosynthetic rate (Teixeira et al., 2022c). In cowpea plants in hydroponic cultivation, Si increased N uptake, improving nodulation and N_2_ fixation (Mali and Aery, 2008a). The improvement in the nutritional status of N with the use of Si was also associated with an improvement in root strength, length, thickness, volume, biomass, number of lateral roots, nodulation, and N fixation (Lux et al., 2020).

The accumulation and use efficiency of P were improved in bean plants after Si was applied via fertigation, probably because this beneficial element improved the action of P on photosynthesis, especially by improving phosphorylation. Foliar P is also a constituent of chemical compounds closely related to photosynthesis (Wang et al., 2018). Moreover, P-deprived plants can decrease light use efficiency, electron transport rates, enzymatic activity in the Calvin cycle, regeneration of ribulose bisphosphate (RuBP), and the fraction of leaf N destined for the photosynthetic machinery (Crous et al., 2017). The physiological effect may also have been favored by the increased availability of P in the soil because the chemical competition between the anions H_2_PO_4_^−^ and silicate (H_3_SiO_4_^−^) by the sorption sites provides a displacement of phosphate into the soil solution, increasing its availability (Etesami and Jeong, 2018). However, it is clear from the correlation analysis that PUE is not an isolated variable decisive in stimulating increased plant tolerance to water deficit scenarios, especially under conditions of severe restriction, because there is probably a more limiting non-nutritional factor in this high-stress situation.

Furthermore, Ca, Mg, and K can adjust the photosynthetic capacity, and these nutrients’ use is improved with the presence of Si. The Ca^2+^ ions provide the terminal acceptor and regulate the flow of photosynthetic electrons, while Mg^2+^ and K^+^ ions have been implicated as light-harvesting counterions in thylakoids and have opposite effects (Battie-Laclau et al., 2014). Increased Mg accumulation positively regulates the photosynthesis of plants under water deficit, especially since this nutrient is involved in the light-dependent reaction by constituting the chlorophyll molecule (Xie et al., 2021). Higher chlorophyll content increases electron transport rates (Battie-Laclau et al., 2014) and then the rates of formation of nicotinamide adenine dinucleotide phosphate (NADPH) and NADP+, which is the terminal acceptor for electron transport (Wang et al., 2018).

It is worth highlighting the fact that in this work, chloride (Cl^−^) levels were not evaluated, but given the relationships of this micronutrient in osmotic adjustment, as well as the fact that it is the accompanying ion of K^+^ in most silicate sources, future research should address the determination of this important anion due to recent discoveries of Cl^−^ as a beneficial macronutrient (Franco-Navarro et al., 2016; Geilfus, 2018; Colmenero-Flores et al., 2019). Also, it is important to mention that even if Cl^−^ was not applied in K0 treatments, plants did not show Cl^−^ deficiency symptoms, as plants did not show bronzing, wilting, or chlorosis symptoms (Broyer et al., 1954; Johnson et al., 1957).

We have discovered that Si can promote the efficient absorption and use of macronutrients and micronutrients by the bean plant. This greater efficiency of nutrient use is induced by Si-provided improvements in the plant’s metabolism, which can be proven by the increase in C use efficiency. The nutritional mechanisms associated with the increase in C use efficiency are justified because Si plays a similar role to C in the plant’s leaf structure (Teixeira et al., 2022b). However, the energy cost of including Si in the C chain is lower (Hao et al., 2020).

Our findings indicate that Si mitigates the damage caused by different levels of water deficiency through a nutritional mitigation mechanism, as there was better absorption of nutrients combined with an increase in nutritional efficiency. It resulted in an increase in the biomass production of the bean plant, confirming this study’s hypothesis. Therefore, this study strengthens the thesis that the effect of Si benefits the physiology of the plant by alleviating water deficit, which is well known in the literature. It provides a significant improvement in the plant’s nutritional status, and this finding should guide Si researchers on this topic to give more value to the plant’s nutritional improvements. However, the beneficial effects are limited to the use of an adequate dose of Si applied through fertigation with a fluid and soluble source. Moreover, the dose is affected by the presence or absence of water deficit. It was also clear that an increase in the severity of water deficit induces the need for a higher dose of this element for optimum growth of the bean plants, possibly because there is a decrease in Si absorption.

Furthermore, it is worth noting that the increase in K absorption, especially with the supply of Si, may give bean plants a greater ability to tolerate water deficit scenarios of different intensities. The relationship between Si and K helps to maintain the plant’s water status (Chen et al., 2016), positively regulating nutrient absorption through the activation of H+-ATPase (Kaya et al., 2006; Mali and Aery, 2008b), intensifying the development of the root system and even affecting the absorption of micronutrients. The synergy between Si and K was evident in this research, affecting the dose of Si to be used in each water availability scenario because, in general, the dose of Si is close to 4 kg/ha without the application of K and the dose close to 8 kg/ha of Si with the application of K resulted in greater nutritional gains and dry mass production. Therefore, we can accept hypothesis ii) that potassium fertilization interferes with the recommended optimum dose of Si. This study’s results draw attention to the fact that it is possible to reduce the water level in the soil without losses in bean growth if this beneficial element can be included in the crop. It was evident in plants with severe water deficit, which had the same dry mass accumulation as moderate water deficit at the doses of Si mentioned above (4 kg/ha of Si in the experiment without K and 8 kg/ha of Si in the experiment with K). Thus, it has significant environmental implications since it is possible to replace “water with Si” in an irrigated cropping system, which will be remarkable in the future, given the scarcity of fresh water (Fan et al., 2021).

Thus, for the first time, the importance of Si application through fertigation as an alternative for efficiently silicating field-irrigated crops is reported. Moreover, this efficient application allowed plants to absorb a sufficient amount of Si, inducing beneficial effects, even in species from the legume group, at relatively low doses. It should expand the use of Si in irrigated agriculture. Therefore, this important role of Si in common bean nutrition observed here has sustainable implications because it should improve the efficiency of water use, given the fact that it is a non-renewable natural resource, and its better use in agriculture is paramount.

## 5 Conclusion

The benefit of silicon in promoting nutrient absorption and utilization indicates tolerance to water deficit in common bean crops. Silicon supplementation in irrigated crops improves water use efficiency and simultaneously increases the crop’s nutritional efficiency. It represents a strategy to achieve sustainable agricultural production of common beans. These findings underscore the potential of silicon to mitigate the damage caused by water deficit through nutritional mechanisms. They highlight the importance of considering both the amount of silicon and water availability in the soil to optimize this crop’s growth and nutritional efficiency.

## Data Availability

The raw data supporting the conclusions of this article will be made available by the authors, without undue reservation.
